# Continuous Separation of Light Olefin/Paraffin Mixtures on ZIF-4 by Pressure Swing Adsorption and Membrane Permeation

**DOI:** 10.3390/molecules23040889

**Published:** 2018-04-11

**Authors:** Maximilian Hovestadt, Sebastian Friebe, Lailah Helmich, Marcus Lange, Jens Möllmer, Roger Gläser, Alexander Mundstock, Martin Hartmann

**Affiliations:** 1 Erlangen Catalysis Resource Center (ECRC), Friedrich-Alexander-Universität Erlangen-Nürnberg (FAU), Egerlandstr. 3, 91058 Erlangen, Germany; maximilian.hovestadt@fau.de; 2 Institute of Physical Chemistry and Electrochemistry, Leibniz University Hannover, Callinstrasse 3A, 30167 Hannover, Germany; sebastian.friebe@pci.uni-hannover.de (S.F.); l.helmich@isfh.de (L.H.); alexander.mundstock@pci.uni-hannover.de (A.M.); 3 Institut für Nichtklassische Chemie e.V. (INC), Permoserstraße 15, 04318 Leipzig, Germany; lange@inc.uni-leipzig.de (M.L.); moellmer@inc.uni-leipzig.de (J.M.); roger.glaeser@uni-leipzig.de (R.G.)

**Keywords:** ZIF-4, olefin/paraffin separation, pressure swing adsorption (PSA), membrane permeation

## Abstract

In this study, two zeolitic imidazolate frameworks (ZIFs) called ZIF-4 and ZIF-zni (zni is the network topology) were characterized by sorption studies regarding their paraffin/olefin separation potential. In particular, equilibrated pure and mixed gas adsorption isotherms of ethane and ethene were recorded at 293 K up to 3 MPa. ZIF-4 exhibits selectivities for ethane in the range of 1.5–3, which is promising for continuous pressure swing adsorption (PSA). ZIF-4 shows high cycle stability with promising separation potential regarding ethane, which results in purification of the more industrial desired olefin. Furthermore, both ZIF materials were implemented in Matrimid to prepare a mixed matrix membrane (MMM) and were used in the continuous separation of a propane/propene mixture. The separation performance of the neat polymer is drastically increased after embedding porous ZIF-4 crystals in the Matrimid matrix, especially at higher feed pressures (3–5 barg). Due to the smaller kinetic diameter of the olefin, the permeability is higher compared to the paraffin.

## 1. Introduction

Several studies have shown the separation capabilities of porous materials for olefin/paraffin separation as a potential green alternative to energy-intensive low temperature distillation [1,2,3]. In particular, pressure swing adsorption and membrane permeation are industrially proven alternative separation processes, but are up till now not used in olefin/paraffin separation due to a lack of selective adsorbents or membranes. An auspicious adsorbent candidate is ZIF-4 [4], which has shown a higher adsorption capacity for paraffin compared to olefin in isotherm measurements as well as a good separation performance in breakthrough experiments for different feed compositions. Thus, ZIF-4 differs from well-known conventional adsorbents such as zeolites [5] and metal–organic frameworks with open metal sites like CPO-27 and Cu_2_(BTC)_3_ [6,7,8]. These materials show stronger interaction with olefin, and the loading is based on the effect described by Dewar, Chatt, and Duncanson [9,10]. A selectivity analogous to ZIF-4 has in principle been reported for similar adsorbents namely ZIF-7 [11], ZIF-8 [7], ZIF-71 [2] FIR-51 [12], UTSA-10 [13], and PCN-250 [14] and several activated carbons [15,16,17]. However, these materials are more expensive in preparation, exhibit an unfavorable separation potential, or are not well studied.

ZIF-4 belongs to the group of so-called zeolitic imidazolate frameworks (ZIF) that consists of zinc metal centers connected to imidazole-based organic linker molecules. Besides ZIF-4, which possesses cag topology, different polymorphs such as ZIF-zni (zni topology), ZIF-1 (BCT), ZIF-3 (DFT), ZIF-6 (GIS), and ZIF-10 (MER) are known [18]. From this group, ZIF-4 possesses the smallest cage opening (ca. 0.2 nm as determined from its crystal structure [18]), while ZIF-zni is supposed to be the densest polymorphs within this combination of metal atoms and linker molecules. Adsorption isotherms of pure gases are typically recorded to render the separation potential of porous materials. While pure gas isotherm measurements only imply an adsorptive selectivity, it is necessary to measure mixed gas isotherms to be able to determine the separation potential by adsorption for dynamic separation processes. 

To improve the separation performance in breakthrough and pressure swing adsorption (PSA) experiments, high-pressure isotherms are beneficial to determine the optimal sorption conditions for dynamic separation. Additionally, a volumetric set-up combined with a gravimetric system allowed us to determine sorption equilibria of binary gas mixtures of ethane and ethene. From these results, we were able to derive the equilibrium selectivity for this sorption process. 

To prove the separation concept with porous materials, continuous separation experiments are indispensable. There are three different instrument-based solutions: the PSA setup, used, e.g., for air separation [19] and hydrogen purification [20], simulated moving bed (SMB) technology, which was tested with zeolite 13X [21], and membrane-assisted separation, which is used for several industry-relevant separation problems such as CO_2_ capture [22,23], hydrocarbon gas separation [24,25], and dewatering [26]. The advantage of these technologies is the ambient separation temperature and the related low energy costs. The PSA technology is well established for separating continuous feed mixtures with adsorber columns filled with porous adsorbents. At least two filled columns are applied alternatively to continuously separate a certain feed mixture. Thereby, the separation is possible due to kinetic effects, differences in adsorption equilibrium, or molecular sieving.

This also applies to membrane-based technologies. The process can be described as a transport across the membrane due to a concentration gradient (Fick’s law). However, for dense (polymer-based) membranes, which transport gas molecules by the solution-diffusion mechanism, the amount adsorbed depends strongly on the ability of the gas to be dissolved in the polymeric matrix, which can be approximated via the condensability of the gas or its molecular size [27]. Mixed matrix membranes (MMMs) are a special case. They consist of a polymer matrix with dispersed fillers (e.g., carbon, ZIFs, and silica). The polymer ensures favorable mechanical properties (flexibility, long-term stability, and reproducibility), while the incorporated additives can either behave as a transport barrier or as separation-active filler. MMMs provide all positive features of both materials and often show increased separation capabilities in comparison to the single materials [28,29,30,31]. 

In this study, the continuous separation potential of ZIF-4 powder for an ethane/ethene gas mixture was tested in a commercial PSA setup. To find the best separation conditions for the continuous setup, high-pressure and mixed adsorption isotherms for ethane and ethene have been recorded. In addition, MMMs with implemented ZIF-4 and ZIF-zni crystals were investigated with respect to their separation potential for propane/propene gas mixtures, which could not be separated using ZIF-4 powder as described in our previous paper [4].

## 2. Results and Discussion

### 2.1. Isotherms

**Figure 1:**
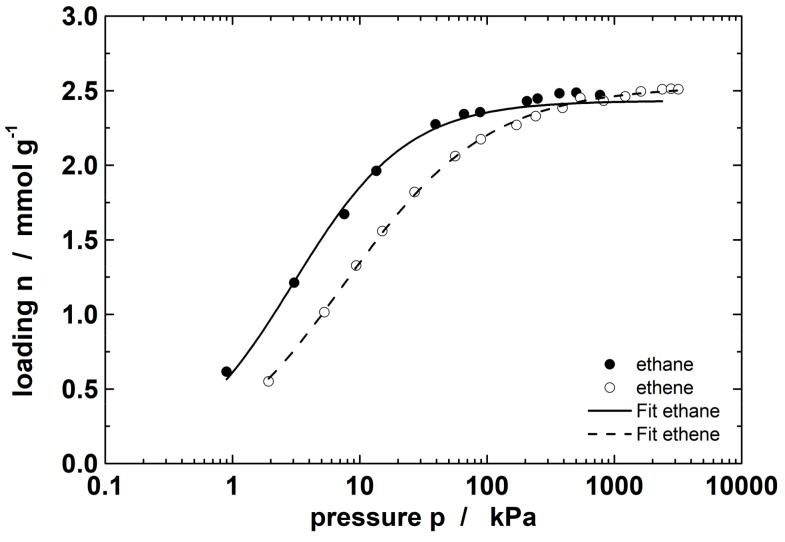
Adsorption isotherm of ethane and ethene at 20 °C on ZIF-4 and the respective fit data from the Toth model.

The equilibrium adsorption isotherms of pure ethane and ethene at 20 °C up to 3 MPa (Figure 1) show the typical type I behavior according the IUPAC isotherm classification [32]. The experimental data were fitted with the adsorption isotherm model of Toth, which describes the experimental data with sufficient accuracy over a wide pressure range.

**Figure 2:**
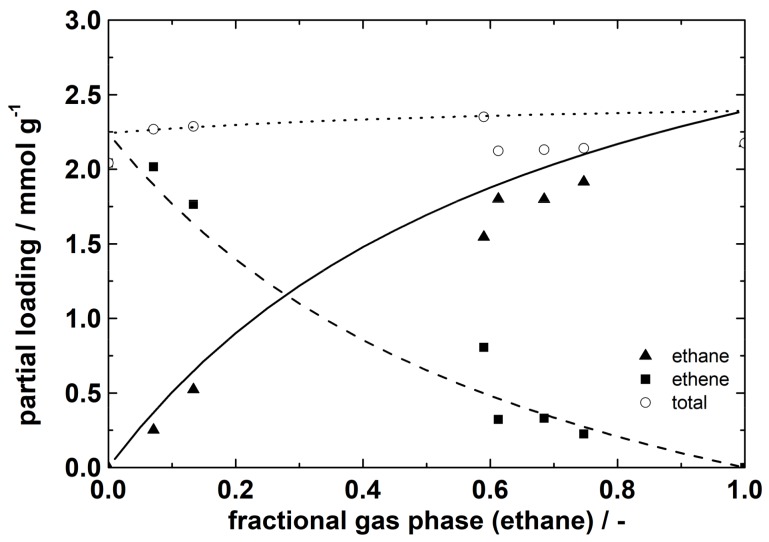
Mixed adsorption equilibria for ethane/ethene at a total pressure of 100 kPa and 20 °C on ZIF-4. Lines demonstrate calculated data from ideal adsorbed solution theory (IAST) based on Toth fit from single gas isotherms.

The presented data show a higher sorption capacity for ethane compared to ethene at lower pressure (0–300 kPa). However, at higher pressure (300–3000 kPa), the loading of ethane reaches a saturation plateau while the loading of ethene still increases. This leads to a decrease in selectivity until a slightly higher loading is found for ethene compared to ethane (for *p* > 500 kPa). This implies that the best separation results should be obtained up to a pressure of about 100 kPa. To demonstrate the ideal behavior for this mixture, we measured the mixed gas adsorption isotherm (Figure 2). 

**Figure 3:**
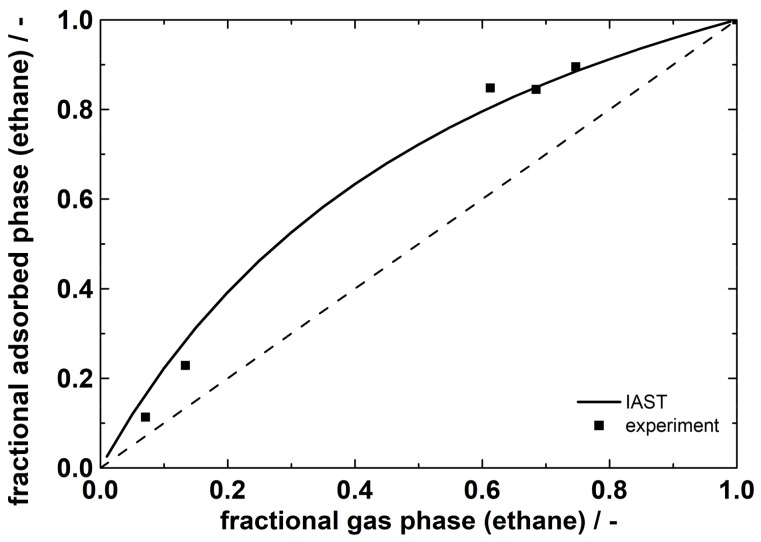
Mixed adsorption equilibria at 20 °C in ZIF-4 for ethane/ethene at a total pressure of 100 kPa. Lines: calculated data by IAST (using Toth).

The plot shows the partial loadings of ethane and ethene as a function of the fractional amount of ethane in the gas phase. The experimental values are compared to the calculated ones from ideal adsorbed solution theory (IAST) based on the Toth fits from single gas isotherms. Because of the good agreement, ideal adsorption behavior can be assumed. From the plot, partial loadings for a certain gas phase fraction can be obtained. In Figure 3, additionally the fractional loading is plotted versus the fractional gas phase of ethane. Both plots illustrate the preferential sorption of ethane on ZIF-4 under the prevailing equilibrium conditions.

In the literature, mixture data of ethane and ethene on common adsorbents like activated carbons (BPL [33], Nuxit-Al [34]) or zeolites (13X [35]) under comparable conditions are reported. In agreement with our study, activated carbons also show an enrichment for ethane. In detail, AC BPL shows an equilibrium selectivity *α* = 1.24 regarding ethane at 301 K and 138 kPa for an ethane/ethene mixture with a ratio of 28/72%. The AC Nuxit-Al has a nearly constant selectivity of *α* = 1.49–1.56 over the entire partial pressure range of ethane at 293 K and 101 kPa. The experimentally observed ethane selectivities for ZIF-4 range from 1.5 to 3, which is higher than the values reported for active carbons. In contrast, zeolites typically exhibit a preferential sorption for ethene as a consequence of favorable adsorption sites for unsaturated hydrocarbons [4]. Kaul et al. [35] reported selectivities of *α* = 4.7–7.4 for the sorption of ethene over ethane on zeolite 13X at 323 K and 138 kPa. Thus, ZIF-4 appears to be a promising adsorbent resulting in a higher retention of ethane for PSA experiments aiming at the continuous separation of olefin/paraffin mixtures. The preferred adsorption of ethane in the absence of strong adsorption sites is triggered by the different critical temperatures of the gases. Sakurovs et al. [36] already showed higher volumetric loadings different gases on coals with increasing critical temperatures. The backdrop of this phenomenon is a faster condensation of a gas as a consequence of its higher boiling point that increases with higher critical temperature. Finally, gas adsorbs more easily with faster condensation [37]. This leads to a stronger adsorption of the paraffin compared to olefin. This behavior changes when the olefin shows stronger interaction with specific sorption sites in a porous adsorbent such as zeolites.

### 2.2. Continuous Separation of Ethane/Ethene

**Figure 4:**
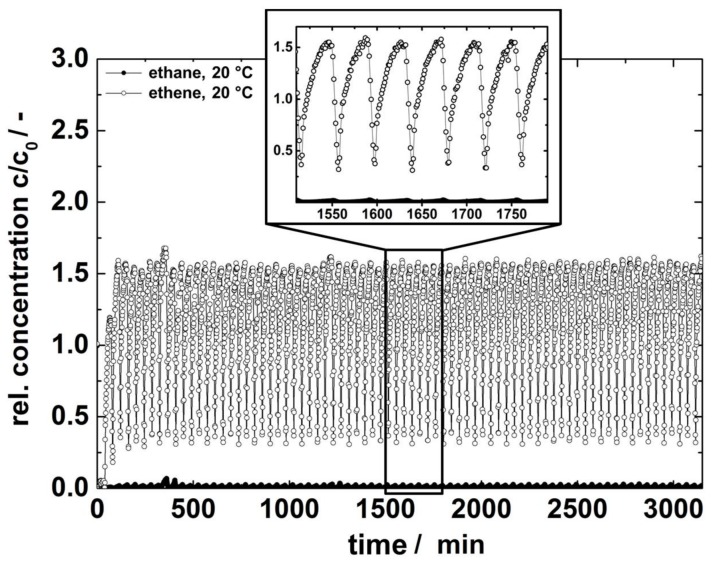
Continuous separation of an equimolar ethane/ethene mixture at ambient pressure and 20 °C.

The continuous separation of an equimolar ethane/ethene mixture with ZIF-4 powder in an PSA setup at 20 °C and ambient pressure is displayed in Figure 4. 

The graph shows the relative concentration of ethane and ethene at the outlet of the PSA setup as a function of the retention time. High broad peaks of ethene (open cycles) with low amounts of ethane (closed cycles) are displayed. For clarity, all data points are connected and a section of the complete measurements is added (cf. Figure 4, inset). The experiment confirmed the results from the mixed-gas isotherm (cf. Section 2.1) and the breakthrough experiments reported in our previous paper showing a higher retention for ethane in line with the single component isotherms [4]. Moreover, the stable separation performance of ZIF-4 for an extended period of time is shown, viz. over 36.5 cycles and 50 h.

In the insert, the switch between both columns is pictured by the concentration drop of ethene. The relative concentration of each peak is related to the degree of dilution by the purge gas nitrogen. By neglecting the amount of nitrogen in the product, the purity of ethene amounts to 99.16%. This renders ZIF-4 as beneficial adsorbents for continuous ethene purification in order to obtain a high level of purity. With the low operation temperature and no need to employ high pressure, the running costs are expected to be low. Therefore, the utilization of ZIF-4 in PSA systems seems to be an economic alternative to ethene purification by low temperature distillation. 

### 2.3. Continuous Separation of Propane/Propene with MMMs

**Figure 5:**
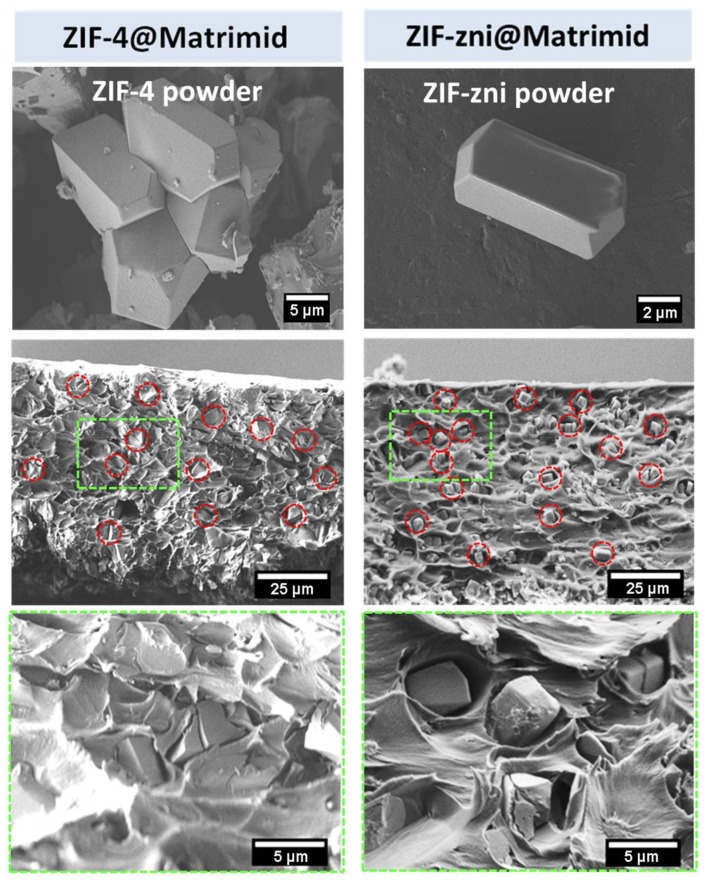
SEM pictures of exemplary MMMs with 10 wt % ZIF-4 (**left**) and ZIF-zni (**right**). Top images show the used powder materials for MMM preparation. Both pictures in the middle are cross sections of the above-mentioned MMMs, which indicate homogeneous distributed filler particles (red circles). Green squares display enlarged areas, shown at the bottom.

The characterization of the applied MMMs regarding the layer thickness and the embedding of the ZIF-4 and ZIF-zni crystals was performed with a scanning electron microscope. The SEM pictures of the MMM cross sections in low and higher magnification are shown in Figure 5.

**Figure 6:**
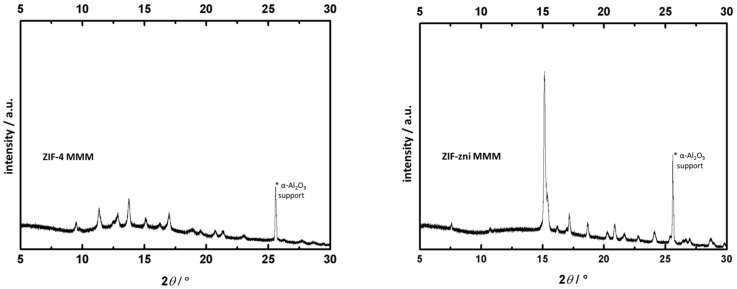
X-ray diffraction patterns of the two different filler based MMMs. (**left**) ZIF-4 particles and (**right**) ZIF-zni particles both embedded in Matrimid.

The images in Figure 5 imply a homogeneous distribution of the crystals in the Matrimid polymer matrix. The crystals seem to be surrounded by voids rather than directly connected to the Matrimid, especially the ZIF-zni particles. These voids may be the result of the preparation for the SEM measurements (breaking) or weak interactions between ZIF-zni and Matrimid as a consequence of a certain incompatibility between the filler and the matrix. The ZIF-4 crystals seem to feature better contact with the Matrimid polymer. Consequently, we expect differences with respect to the gas separation capabilities between the two composite materials not only because of their different porosity but also as a result of their different compatibility with the surrounding polymer. Nevertheless, the SEM results look promising for gas separation measurements with the corresponding MMMs. The detected thickness of the ZIF-4 membrane is 77 µm, while 108 µm are measured for the ZIF-zni membrane. Both ZIFs are embedded in the polymer matrix as evident from their characteristic diffraction lines. Furthermore, the amorphous fraction of the used polymer Matrimid is visible as a smooth bump between 10 and 15° (see Figure 6).

**Figure 7:**
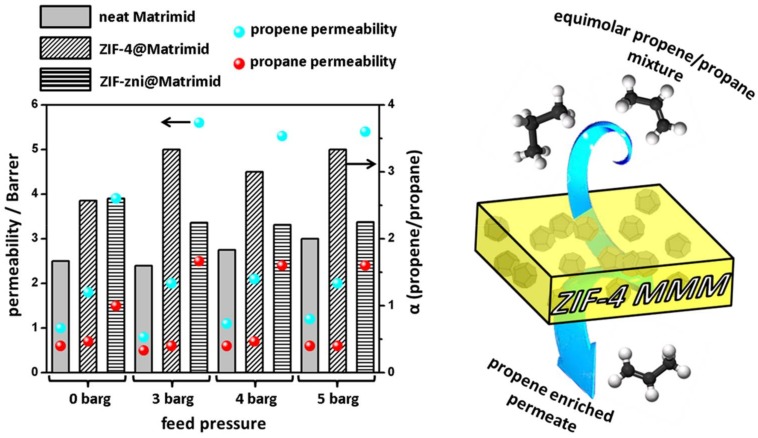
Mixed gas permeabilities for propene and propane as well as separation factors for two MMMs with porous materials (ZIF-4 and ZIF-zni) compared to a neat Matrimid membrane measured at 150 °C (**left**). Schematic demonstration of a separation process with an MMM (**right**).

A feed mixture of propane and propene was used to investigate the influence of the porous adsorbents ZIF-4 and ZIF-zni as filler in MMMs in comparison to the neat polymer material Matrimid. The measured permeability as well as the calculated separation factor (α_propene/propane_) for both gases on MMMs with ZIF-4, ZIF-zni and neat Matrimid is displayed in Figure 7.

For porous materials assembled in MMMs, the mixed gas permeabilities as well as the separation factor is generally higher compared to the neat Matrimid. The neat Matrimid membrane shows selectivities around 1.5 and propene permeabilities around 1 Barrer at 0 barg. With increasing feed pressure, both remain more or less constant. By embedding porous ZIF-4 crystals in the Matrimid matrix, the separation performance of the neat polymer is drastically increased, especially at higher feed pressures (3–5 barg). Under these conditions, both the propene permeability and the separation factor are almost doubled in comparison with the neat Matrimid. When adding the denser phase ZIF-zni, the separation factor is slightly enhanced in comparison to the neat Matrimid (1.5 to ≈2.3). In contrary to this, the propene permeability is almost six times higher compared to the neat polymer film. These high permeabilities are most likely a consequence of the void-rich ZIF-zni MMM, since the contact between the filler and the polymer phase is weak. The permeation results are in complete accordance with the SEM results in Figure 7. For the same reason, the separation factor is nearly the same compared to the neat Matrimid membrane, because ZIF-zni barely contributes to the separation capabilities.

## 3. Experimental Section

### 3.1. Porous Adsorbents

#### 3.1.1. ZIF-4

ZIF-4 was synthesized as described elsewhere [4]. In a typical synthesis, 7.2 g of zinc nitrate hexahydrate (Sigma-Aldrich, Steinheim, Germany, purity 98%) and 4.8 g of imidazole (Acros Organics, NJ, US, purity 99.5%) were dissolved in 240 mL of dimethylformamide (Honeywell, Seelze, Germany, purity 98–99%), heated up to 130 °C and stirred for 48 h. 

#### 3.1.2. ZIF-Zni

The synthesis for ZIF-zni is based on the pathway of Bennett et al. [38] with small modifications developed in our laboratory. Therefore, 0.67 g of zinc nitrate hexahydrate (Alfa Aesar, Karlsruhe, Germany, purity 99%) and 0.5 g of imidazole (Sigma Aldrich, Steinheim, Germany, purity ≥ 99.5%) were each dissolved in 50 mL of methanol (Sigma Aldrich, Steinheim, Germany, purity ≥ 99.8%). Additionally, both solutions were mixed together under brief stirring in a Schott flask, followed by a rest period overnight and a heating period at 100 °C for 7 h. After completion of the synthesis, two cooling procedures are applied, viz. at room temperature and by an ice bath. Both materials are washed twice with methanol and dried at 70 °C in an oven. 

### 3.2. Mixed Matrix Membranes

Prior to the preparation of the mixed matrix membranes, the required ZIF-4 and ZIF-zni crystals were synthesized as described in Section 3.1. After synthesis, the powder materials were activated in a vacuum oven at 100 °C and 50 mbar overnight. For the preparation of the MMMs, 10 mg of the obtained crystals were suspended with 90 mg of Matrimid 5218 US (Huntsman, The Woodlands, TX, USA) in 1 ml of dichloromethane (DCM) (Sigma Aldrich, purity ≥ 99.9%) under stirring overnight. The preparation was done inside a glove box to ensure inert gas atmosphere. The dispersion was first thickened to 400 µL under inert nitrogen gas atmosphere and then cast onto a porous α-Al_2_O_3_ support with a pore diameter of 1.8 µm. The hardening procedure occurs under a DCM atmosphere. After evaporation of the solvent in Petri dishes, the MMMs were activated in the measurement cell at 150 °C and under a nitrogen flow to remove residual solvent (usually 24 h under a constant nitrogen flow). For pictures of cross sections by scanning electron microscopy (JSM-6700F from JEOL, Tokyo, Japan), the MMMs were broken into two pieces and fixed on a brass support with carbon pads. Subsequently, they were sputtered with carbon for electric conductivity.

### 3.3. Apparatus for High-Pressure and Mixed Isotherms

The pure gas equilibrium isotherms of ethane and ethene at 20 °C were measured with a gravimetric-magnetic suspension balance (MSB) from Rubotherm, Bochum, Germany (resolution 10 µg). For the measurements, 0.3 g of ZIF-4 were activated at 200 °C for at least 12 h in vacuum. The respective gas was dosed into the sample chamber. The weight as well as the gas pressure was recorded. The equilibrium state was assumed when the weight change was <10 µg and pressure was constant for 10 min. For further measurement points, the gas pressure was stepwise elevated.

**Figure 8:**
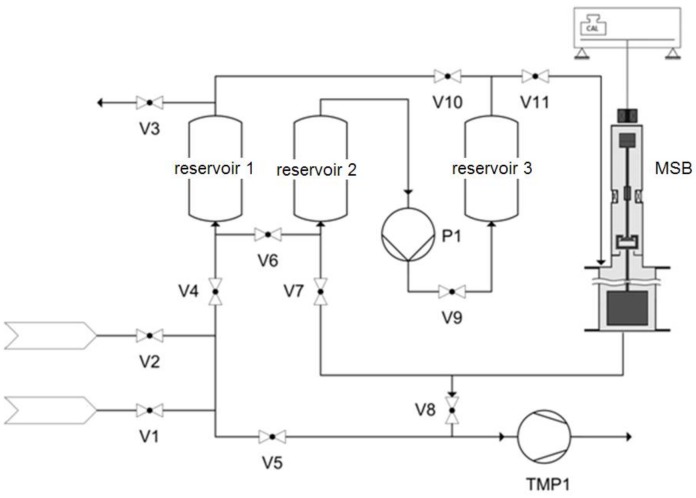
Schematic view of a manometric-gravimetric adsorption setup.

For the mixed gas equilibrium isotherm, a manometric-gravimetric setup was employed. This is assembled by an MSB, which is integrated in a manometric setup (see Figure 8).

The gas phase composition that is necessary for balancing the manometry was analyzed with the gas chromatograph HP5890 series II from Hewlett-Packard equipped with a flame ionization detector (FID). For the measurement, ethane was first submitted to the measuring chamber until equilibrium (weight change <10 µg and constant pressure for 10 min) was reached at 100 kPa. Subsequently, portions of ethene were dosed stepwise at 100 kPa total pressure to reduce the amount of ethane in the gas phase and to enforce a partial desorption of ethane from ZIF-4. The equilibrium of the mixed gases has been calculated from the mass balance given by pairs of pressure, temperature, gas phase composition, and loading before and after the dosing of ethene to the measuring chamber. For mixed gas adsorption measurements, an amount of 1.5 g of activated ZIF-4 was used.

The selectivity was calculated from experimental data (cf. Figure 2) by using the following equation:

$$\text{equilibrium}\;\text{selectivity}\;\propto \;=\;\frac{\left(\frac{x_{1}}{y_{1}}\right)}{\left(\frac{x_{2}}{y_{2}}\right)}\;1\;=\;\text{ethane}\;\text{and}\;2\;=\;\text{ethane}.$$

The selectivity for activated carbons and zeolites are estimated from a set of experimental data taken from the literature using the same equation. We have added this information to the experimental section.

### 3.4. The Pressure Swing Adsorption Setup

**Figure 9:**
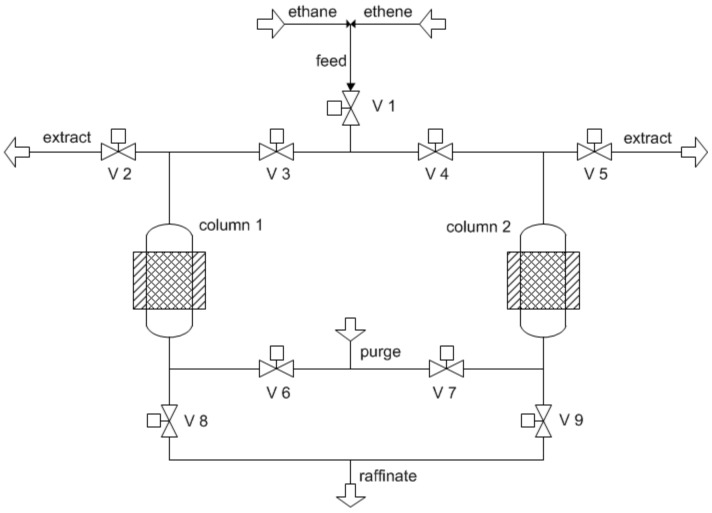
Simplified schematic view of the applied two-bed pressure swing adsorption (PSA) setup.

For the continuous separation of ethane and ethene mixtures, the lab scale pressure swing adsorption setup PSA-300LC from L&C Science and Technology, Hialeah, FL, USA) was used. A thermostat with Peltier element was employed to keep a constant temperature of 20 °C in both adsorber columns (Figure 9). 

These are filled with 1.164 g (column 1) and 1.188 g (column 2) of ZIF-4 with a particle size between 106 and 250 µm. Both packings were ex situ activated in a vacuum oven at 150 °C for 20 h. The inner volume of one column is ca. 2.21 cm³. For continuous separation, an operation mode similar to the Skarstrom cycle [39,40] was used. The experiments were conducted at ambient feed pressure, no pressurization or blowdown step was necessary. 

One cycle consists of the adsorption and purging step of the first column and the purging and adsorption step of the second column. The adsorption step of the first column runs parallel to the purging step of the second column. This cycle can be repeated ad libitum and defines the length of the separation experiment. 

The feed (${\overset{\cdot }{V}}_{\mathrm{feed}}$ = 2 mL_N_/min), an equimolar mixture of ethane and ethene, is loaded and separated during the adsorption step. For the purging step, nitrogen (${\overset{\cdot }{V}}_{\mathrm{purge}}$ = 150 mL_N_/min) was used to regenerate the loaded column in counter current to the feed. 

By purging with a third gas and running the process at ambient pressure, this mode of operation is sometimes rather called concentration swing adsorption (CSA). This expression is related to the process described by Rao et al. for separating azeotropic liquid mixtures where a third component was used for purging [41]. The cycle time is 82 min, which is the sum of the separation times for both columns, viz. 40 min for Column 1 and 42 min for Column 2. The different adsorption intervals are related to the different batting of the columns. The time of the adsorption step is determined from mixed breakthrough experiments for each column. The applied separation time is related to the breakthrough times of ethane. For downstream analysis of the gas mixture at the adsorber outlet, a gas chromatograph GC-456 Scion from Bruker equipped with an FID was employed.

### 3.5. Membrane Setup

**Figure 10:**
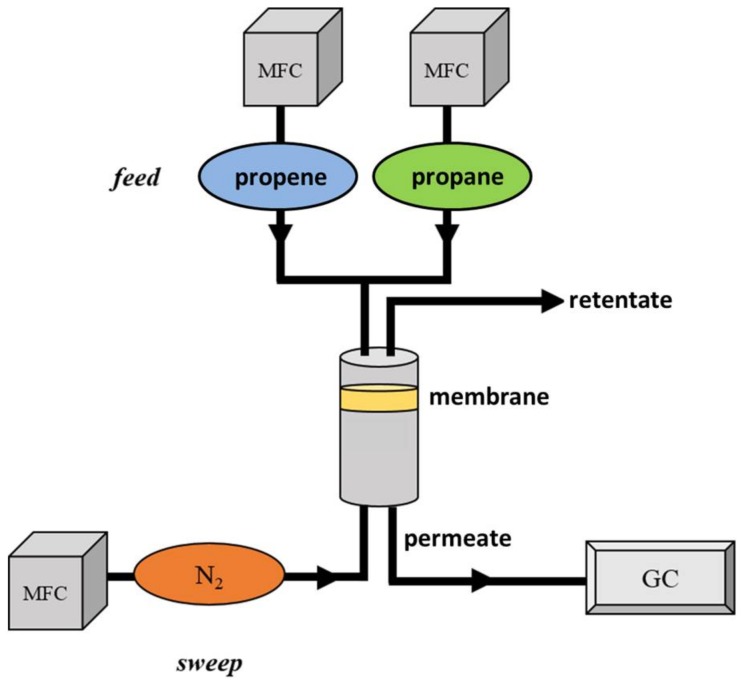
Schematic view of the experimental setup according to Wicke Kallenbach used for permeation measurements with mixed matrix membranes (MMMs) and binary propane/propene mixtures.

For the permeation measurements, a home-built apparatus (displayed in Figure 10) based on the idea of Wicke Kallenbach was used. An equimolar mixture of propane and propene (${\overset{\cdot }{V}}_{\mathrm{feed}}$ = 60 mL_N_/min) is transported to the membrane in the permeation cell. The molecules passing the membrane are called permeate and the retained molecules leave the permeation cell as retentate. To preserve a high driving force and to enable analysis, the permeate is swept with nitrogen (${\overset{\cdot }{V}}_{\mathrm{sweep}}$ = 1 mL_N_/min) toward a gas chromatograph (Agilent Technologies 7890 B, Waldbronn, Germany). The permeation experiment was carried out at a constant temperature of 150 °C and at different feed pressures (0 barg, 3 barg, 4 barg, and 5 barg). Measurements were carried out until the equilibrium was reached, which was usually achieved after 24 h of continuous testing.

## 4. Conclusions

The sorption and separation potential of ZIF-4 powder as well as ZIF-4 and ZIF-zni crystals embedded in Matrimid membranes were studied regarding paraffin and olefin separation using different experimental techniques, in particular gravimetry–manometry, pressure swing adsorption, and membrane permeation.

Adsorption isotherms of ethane and ethene at 20 °C showed a higher loading of ethane compared to ethene in the low pressure range, which results in a higher retention of unwanted ethane. For higher pressure (>200 kPa), the separation performance is reduced due to the smaller difference in loading for both gases. Finally, the loading of ethene exceeds the values of ethane, which implies a higher retention of the requested gas and is adverse for its purification.

The mixed isotherms for ethane and ethene showed ideal behavior, which allows to predict further equilibrium data for mixtures with ideal adsorption solution theory based on pure component isotherms. 

The continuous separation of equimolar ethane/ethene mixtures was successfully demonstrated with ZIF-4 powder in the lab scale pressure swing adsorption setup. Over a specific period of 50 h, the separation performance does not decline, which is related to the stable adsorption behavior of ZIF-4 and the proper regeneration during the purging step. Therefore, the separation is more influenced by static adsorption than kinetic effects. 

The embedding of ZIF-4 and ZIF-zni crystals in the polymer matrix Matrimid was successful and showed beneficial properties of the resulting MMM with respect to the permeability of propane and propene compared to the neat Matrimid membrane. The best separation factor of propane and propene was achieved with ZIF-4 crystals in MMMs that doubled the value of the neat Matrimid. Separation capabilities might be further improved in future studies by increasing the interaction between the filler and the matrix.
